# Compound *Taxus chinensis* Capsule Combined with Chemotherapy for Non-Small-Cell Lung Cancer: A PRISMA-Compliant Systematic Review and Meta-Analysis of Randomized Controlled Trials

**DOI:** 10.1155/2021/9535061

**Published:** 2021-12-16

**Authors:** Keshuai Li, Haibo Cheng, Weixing Shen, Elaine Lai-Han Leung, Shao Le, Lili Yu, Han Xie, Xinbing Sui, Xiaoming Zhu, Qibiao Wu

**Affiliations:** ^1^State Key Laboratory of Quality Research in Chinese Medicine, Macau University of Science and Technology, Avenida Wai Long, Taipa, Macau, China; ^2^Faculty of Chinese Medicine, Macau University of Science and Technology, Avenida Wai Long, Taipa, Macau, China; ^3^The First Clinical Medical College of Nanjing University of Chinese Medicine, Jiangsu Collaborative Innovation Center of Traditional Chinese Medicine Prevention and Treatment of Tumor, Nanjing, Jiangsu, China; ^4^The First Hospital of Hunan University of Chinese Medicine, Changsha, Hunan, China; ^5^College of Pharmacy and Department of Medical Oncology, The Affiliated Hospital of Hangzhou Normal University, School of Medicine, Hangzhou Normal University, Hangzhou, Zhejiang, China; ^6^Guangdong-Hong Kong-Macao Joint Laboratory for Contaminants Exposure and Health, Guangzhou, Guangdong, China; ^7^Zhuhai MUST Science and Technology Research Institute, Zhuhai, Guangdong, China

## Abstract

**Background:**

Compound *Taxus chinensis* capsule (CTCC), an antitumor Chinese patent medicine, has been commonly prescribed as an adjunctive agent to chemotherapy for the management of non-small-cell lung cancer (NSCLC); however, the effects of CTCC added to chemotherapy for NSCLC patients have never been comprehensively evaluated or summarized.

**Purpose:**

To assess the synergistic effects of CTCC and chemotherapy on NSCLC. *Study Design*. Evidence-based study, systematic review, and quantitative meta-analysis.

**Methods:**

This systematic review and meta-analysis was implemented in accordance with the PRISMA (Preferred Reported Items for Systematic Review and Meta-Analysis) guidelines. Eight databases including China National Knowledge Infrastructure, SINOMED, China Biomedical Literature Database, Wanfang Database, VIP, PubMed, Cochrane Library, and EMBASE were searched for relevant RCTs from their inception until May 24, 2021, and hand-searching was also carried out to identify additional studies. All randomized controlled trials (RCTs) that compared CTCC combined with chemotherapy versus chemotherapy alone were included in our study. The Cochrane Risk-of-Bias tool was used to determine the risk of bias and methodological quality of the included RCTs. Review Manager 5.3 software was used for comprehensive analysis. The primary outcome measure for this study was the disease control rate (DCR), and the secondary outcomes included the objective response rate (ORR), adverse reactions, and quality of life (QOL).

**Results:**

Six RCTs with a total sample size of 410 were finally included. The pooled data showed that, compared with chemotherapy alone, CTCC combined with chemotherapy significantly improved DCR (RR = 1.15, 95% CI: 1.06–1.25, *P* = 0.006), ORR (RR = 1.38, 95% CI: 1.18–1.63, *P* *<* 0.00001), and QOL (MD = 8.69, 95% CI: 7.26–10.13, *P* < 0.006) and reduced the incidence of total adverse reactions (RR = 0.48, 95% CI: 0.38–0.60, *P* < 0.00001). The subgroup analyses indicated that CTCC plus chemotherapy significantly improved gastrointestinal reactions (*P* = 0.004), leukopenia (*P* = 0.0009), thrombocytopenia (*P* = 0.01), rash (*P* = 0.002), and fever (*P* = 0.007).

**Conclusion:**

Based on the available evidence, compared with chemotherapy alone, CTCC used as an adjunctive agent to chemotherapy for NSCLC can improve the clinical efficacy and quality of life and decrease the likelihood of adverse reactions, suggesting that CTCC might be an effective and safe adjunctive medicine to chemotherapy for NSCLC. However, considering the relatively small sample size and the inherent imperfections of the included randomized controlled trials, more high-quality clinical trials with longer follow-up time are needed to further assess the efficacy and safety of this combined treatment regimen.

## 1. Introduction

According to the Global Cancer 2020 Statistics (GLOBOCAN 2020) [1], there were 19.3 million new cases of malignant tumors worldwide and approximately 10 million deaths from malignant tumors worldwide. By 2020, there were about 1.8 million lung carcinoma deaths which accounted for 18% of all carcinoma death [2]. Lung carcinoma is still the main cause of cancer-related deaths, and non-small-cell lung cancer (NSCLC) is the most common, accounting for about 80−90% of all cases of lung carcinomas [3]. Surgical operation is currently the first choice for the remedy of lung cancer, but it often brings heavy pain to patients, and most patients are not suitable for surgery once they are diagnosed with this disease. Although immunotherapy and targeted therapy have remarkably improved the clinical outcomes of patients with NSCLC, chemotherapy is still an irreplaceable treatment method for many patients who lack specific biomarkers or access to these therapies. Because chemotherapy is often associated with more adverse reactions, such as myelotoxicity and gastrointestinal symptoms, many patients cannot tolerate it. The quality of life (QOL) in lung cancer patients undergoing chemotherapy is usually poor. Therefore, it is still a pressing need to look for optimal lung cancer treatment which not only effectively improves the clinical treatment efficacy but also reduces the physical, psychological, and economic burden of patients.

In recent decades, a lot of clinical studies have proved that traditional Chinese medicine plus chemotherapy has some advantages, as Chinese medicine might increase the response to chemotherapy and alleviate the adverse reactions of patients with NSCLC [4–6]. Compound *Taxus chinensis* capsule (CTCC) is an antitumor drug and has been increasingly prescribed as an adjunctive treatment to chemotherapy for the management of NSCLC. A few clinical trials have suggested that this combination treatment might bring benefits to the patients with NSCLC. However, due to the small sample sizes, most of the clinical studies provided insufficient evidence and had only borderline statistical power; therefore, a meta-analysis was needed to combine these clinical trial data, thus increasing the sample size and the power to obtain a more precise and stable estimate of the effect of CTCC plus chemotherapy on NSCLC patients, which has never been performed before. This evidence-based study was carried out to assess the effectiveness and safety of CTCC in combination with chemotherapy for NSCLC patients, aiming to obtain important evidence for the clinical application of this combined treatment regimen.

## 2. Materials and Methods

Following the PRISMA (Preferred Reported Items for Systematic Review and Meta-analysis) guidelines [7], we performed this systematic review and meta-analysis of RCTs that compared CTCC plus chemotherapy versus chemotherapy for NSCLC.

### 2.1. Criteria for Selection of Studies

#### 2.1.1. Types of the Included Clinical Studies

All RCTs comparing CTCC combined with chemotherapy versus chemotherapy alone were evaluated for inclusion in the present study.

#### 2.1.2. Types of the Included Participants

All the included participants were diagnosed with NSCLC by pathology or histology. The patients with other types or unknown types of lung carcinoma or other tumors were excluded. Gender, age, staging, and sample size were not limited.

### 2.2. Types of Interventions

The control group was treated with chemotherapy alone, and the experimental group was treated with CTCC plus chemotherapy.

### 2.3. Types of Outcome Measures

In this study, the primary outcome measure was the disease control rate (DCR), and the secondary outcomes included the objective response rate (ORR), adverse reactions, and quality of life (Karnofsky score).

Following the WHO criteria [8, 9], the common indicators for reporting the results of cancer treatment included progressive disease (PD), stable disease (SD) or no change (NC), partial response (PR), and complete response (CR). DCR and ORR are defined as DCR = (CR + PR + SD)/total × 100%, ORR = (CR + PR)/total × 100%. Both outcomes are commonly used for evaluating the response to cancer treatment.

KPS is a common index to evaluate the quality of life. The higher the score, the better the quality of life. Adverse reactions refer to the chemotherapy-induced adverse drug reactions which include gastrointestinal reactions, leukopenia, thrombocytopenia, rash, and fever.

### 2.4. Criteria for Exclusion of Studies

The criteria for exclusion of studies are (a) negative diagnosis of NSCLC; (b) not RCT study design; (c) without CTCC treatment; (d) chemotherapy regimen being not clear; (d) duplicate publications; (e) inability to obtain full-text articles or extract data; and (f) animal experiments, theoretical studies, or reviews.

### 2.5. Retrieval Method

We conducted a comprehensive search of the China Biomedical Literature Database, China National Knowledge Infrastructure, SINOMED, Wanfang Database, VIP, PubMed, EMBASE, and Cochrane Library to collect all potential RCTs of CCTC plus chemotherapy for the treatment of NSCLC. Hand searching was also carried out to identify more additional studies that were not included in the common electronic databases. The search time was from the inception of the databases to May 24, 2021.

Two independent reviewers (KS Li and HB Cheng) searched the electronic databases. The Chinese databases were searched using the following terms: (“Fufanghongdonsan” (compound *Taxus chinensis*)' OR “Fufanghongdonsan jiaonang” (compound *Taxus chinensis* capsule) AND “feiai” (lung cancer) OR “feixiaoxibaofeiai” (non-small-cell-lung cancer OR carcinoma) AND “hualiao” (chemotherapy). The following terms were used to retrieve studies in the English databases: ((“compound Hongdoushan capsule” OR “compound *Taxus chinensis* capsule”) AND (“non-small-cell lung cancer” OR “non-small-cell lung carcinoma,” “non-small-cell lung carcinomas” OR “non-small-cell lung” OR “lung cancer, non-small-cell” OR “lung carcinomas, non-small-cell” OR “lung carcinomas, non-small-cell” OR “cancer, non-small cell lung” OR “carcinoma, non-small cell lung”) AND (“chemotherapy” OR “chemotherapeutics” OR “chemotherapeutic agents” OR “chemotherapeutic drugs”)). A search strategy for PubMed with PICO model was presented as an example in the Appendix.

### 2.6. Screening and Evaluation of Literature Data

The studies were screened and summarized, and the information was extracted and cross-checked by two independent researchers (KS Li and HB Cheng). If there was a disagreement, a third reviewer (QB Wu) would be invited to discuss and solve the disagreement. Data extraction included (a) the headline of the paper, the lead author, and issuing time; (b) the total RCT sample, the sample size, and the interventions in either group; (c) outcome measures including DCR, ORR, KPS, and adverse reactions.

The Cochrane Risk-of-Bias tool was utilized to assess the methodological quality of all included RCTs and the risk of bias across the studies according to the following items: the random sequence generation, the allocation concealment, the blinding methods, the incomplete outcome data, the selective outcome reporting, and other bias sources. The bias risk was graded as high risk of bias (-), unclear risk of bias (?), and low risk of bias (+).

### 2.7. Statistical Analysis Methods

RevMan 5.4.1 software was applied for this meta-analysis. The continuous data were represented by weighted mean difference (WMD) or standardized mean difference (SMD) with 95% CI. The dichotomous data were represented by risk ratio (RR), risk difference (RD), or odds ratio (OR) with 95% confidence intervals (CI). The chi-square and *I*^2^ tests were used to evaluate the potential heterogeneity. When there was statistical homogeneity between studies (*I*^2^ < 50%, *P* > 0.1), meta-analysis was carried out using the fixed-effects model. If there was substantial heterogeneity (*I*^*2*^ ≥ 50%, *P* *<* 0.1), meta-analysis was performed using the random-effects model, and heterogeneity was further addressed by sensitivity analysis, subgroup analysis, etc. [10, 11].

### 2.8. Risk of Bias across Trials

When a meta-analysis contains at least 10 clinical trials, the potential risk of bias across trials is examined by the funnel plots and Egger's test [12, 13].

## 3. Results

### 3.1. Process and Results of Literature Screening

**Figure 1:**
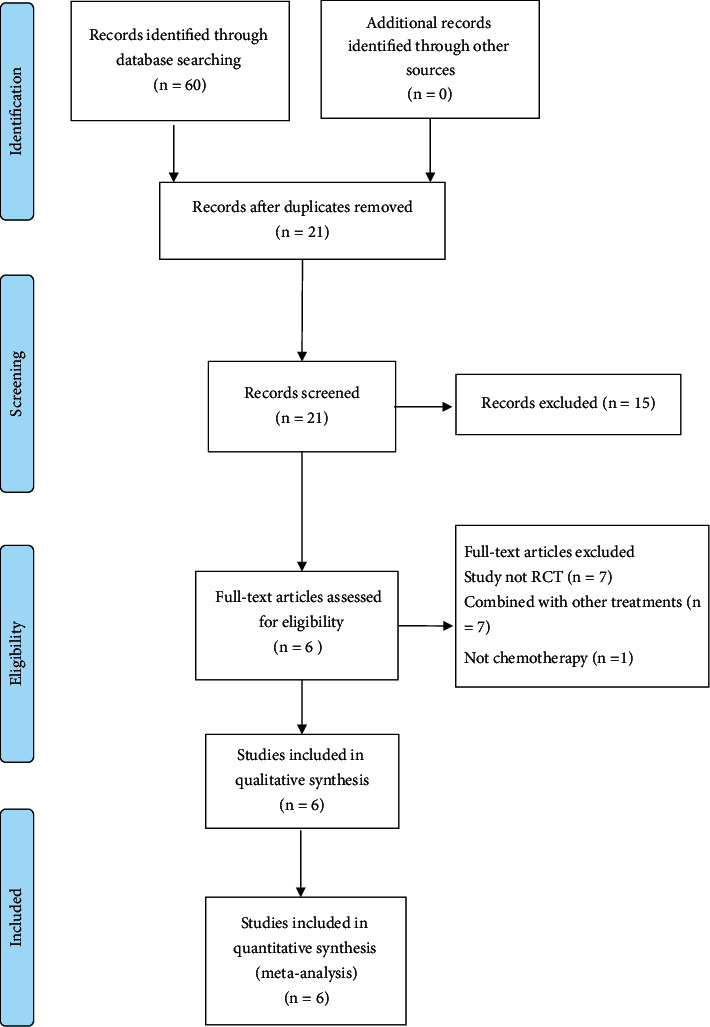
Flowchart of the PRISMA literature search. RCT = randomized controlled trial; PRISMA = preferred reporting items for systematic reviews and meta-analysis.

A total of 60 publications were obtained through preliminary search in the database and other Supplementary Materials, 16 articles from the Wanfang database, 12 articles from CNKI, 17 articles from China Biomedical Literature Database, and 15 articles from VIP Database. After screening, 6 RCTs [14–19] were selected.  Figure 1 shows the specific results.

### 3.2. Main Features of the Included RCTs

Six RCTs with a total sample size of 410 were finally included. The experimental and control groups have equal samples sizes. The experimental group was treated with CTCC combined plus chemotherapeutic drugs, and the patients in the control group received chemotherapy alone. The main features of the six RCTs included in our study are shown in Table 1.

### 3.3. Quality of the Included RCTs

**Figure 2:**
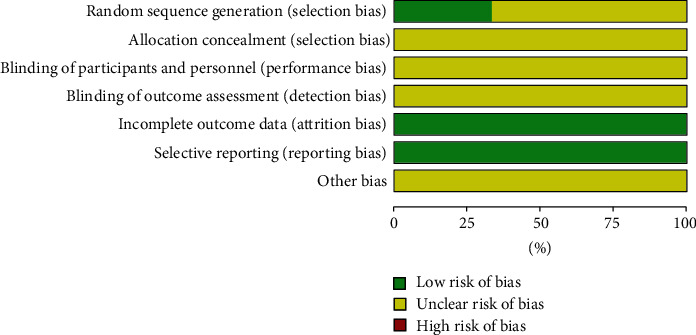
Graph of risk of bias.

**Figure 3:**
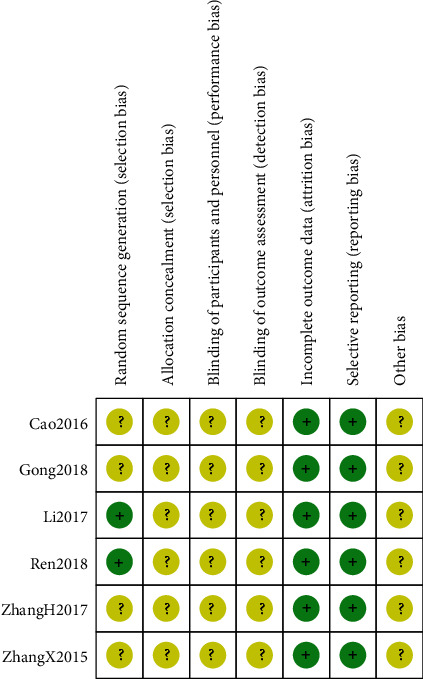
Summary of risk of bias.

Among the 6 RCTs, two trials [15, 18] clearly described the randomization method (the random number table method) in terms of random sequence generation. Randomization was used in the remaining four RCT [14, 16, 17, 19], but they did not clearly describe the randomization method. In most studies, the allocation concealment, the blindness of the participants, the age of each person, the evaluation of the results, and the selective reporting were not clear. In all the included RCTs, the data were intact. Other risk of bias was unclear. The specific results are shown in Figures 2 and 3.

### 3.4. Meta-Analysis Results

The meta-analysis results are shown in Table 2.

#### 3.4.1. DCR and ORR

**Figure 4:**
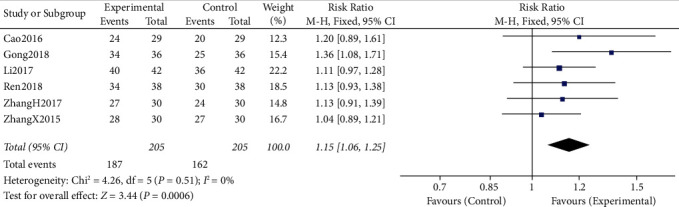
Forest plots representing the pooled risk ratio of the DCR between the experimental and control groups.

**Figure 5:**
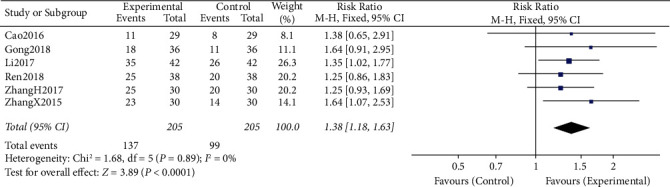
Forest plots representing the pooled risk ratio of the ORR between the experimental and control groups.

All the six included RCTs reported the short-term clinical efficacy of CTCC in combination with chemotherapy for the management of NSCLC (DCR, ORR). Meta-analysis results indicated that, compared with chemotherapy alone, CTCC plus chemotherapy significantly improved the DCR (RR = 1.15, 95% CI: 1.06–1.25, *P* = 0.006) and ORR (RR = 1.38, 95% CI: 1.18–1.63, *P* < 0.00001). There was statistical homogeneity for these two outcomes (both *I*^*2*^ = 0%), a fixed-effects model was adopted to calculate pooled effect estimates (Figures 4 and 5).

#### 3.4.2. Quality of Life (KPS Health Score)

**Figure 6:**
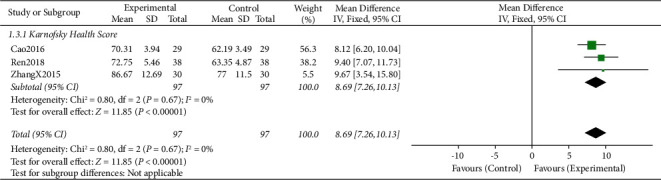
Forest plots representing the pooled mean difference of the QOL between the experimental and control groups.

There were 3 RCTs [14, 18, 19] comparing the KPS scores of patients treated with CTCC plus chemotherapy vs. chemotherapy alone. CTCC plus chemotherapy was superior to chemotherapy alone for the enhancement of QOL (MD = 8.69, 95% CI: 7.26–10.13, *P* < 0.006, *I*^*2*^ = 0). The difference between the two groups was statistically significant (*P* < 0.05) (Figure 6).

### 3.5. Adverse Reactions

**Figure 7:**
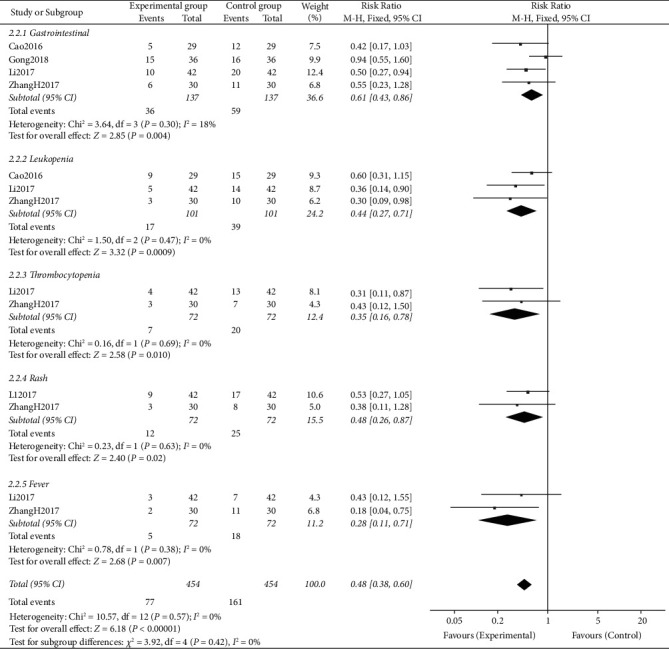
Forest plots representing the pooled risk ratio of the incidence of toxicities between the experimental and control groups.

Four studies [14–17] investigated and reported the adverse reactions of CTCC combined with chemotherapy. The results of meta-analysis suggested that, compared with chemotherapy alone, CTCC added to chemotherapy significantly reduced the total occurrence of adverse reactions (RR = 0.48, 95% CI: 0.38–0.60, *P* < 0.00001) (Figure 7).

The subgroup analyses showed that CTCC plus chemotherapy significantly alleviated gastrointestinal reactions (RR = 0.61, 95% CI: 0.43–0.86, *P* = 0.004, *I*^*2*^ = 18%), thrombocytopenia (RR = 0.35, 95% CI: 0.16–0.78, *P* *=* 0.01, *I*^*2*^ = 0%), leukopenia (RR = 0.44, 95% CI: 0.27–0.71, *P* = 0.0009, *I*^*2*^ = 0%), fever (RR = 0.28, 95% CI: 0.11–0.71, *P* = 0.007, *I*^*2*^ = 0%), and rash (RR = 0.48, 95% CI: 0.26–0.87, *P* = 0.002, *I*^*2*^ = 0%) (Figure 7).

### 3.6. Publication Bias

Because the total number of the included trials was less than 10, Egger's test and funnel plots were not implemented to assess the potential risk of bias across trials.

### 3.7. Subgroup and Sensitivity Analyses

In our study, there was overall homogeneity among the included RCTs. DCR was defined as the primary outcome measure of our study. The pooled data indicated that, compared with the chemotherapy alone, CTCC in combination with chemotherapy significantly increased the DCR of sufferers with NSCLC (RR = 1.15, 95% CI: 1.06–1.25, *P* = 0.006). The results remained robust when subgroup and sensitivity analyses were carried out based on the sample size (≥30 participants per group) (RR = 1.19, 95% CI: 1.07–1.32, *P* = 0.002), chemotherapy regimen (GP (RR = 1.19, 95% CI: 1.07–1.32, *P* = 0.002); non-GP regimens (RR = 1.21, 95% CI: 1.07–1.38, *P* = 0.003)), or the year of publication (published in the last 5 years) (RR = 1.17, 95% CI: 1.07–1.29, *P* = 0.001).

## 4. Discussion

As one of the widely used antitumor drugs in China, CTCC has been broadly utilized as an adjunctive drug treatment to chemotherapy for the management of NSCLC, but the effectiveness and safety of CTCC combined with chemotherapy for NSCLC have never been systematically assessed. The current meta-analysis demonstrates for the first time the synergic effects of CTCC and chemotherapy on the clinical outcomes of NSCLC patients. The findings of this evidence-based study clearly demonstrate that this combination therapy significantly improves several important outcomes such as tumor responses, QOL, and toxicities, indicating that this combination therapy might bring benefits to the patients with NSCLC.

According to Chinese medicine theory, the treatment principle for lung cancer is strengthening the vital Qi to eliminate pathogenic factors. CTCC is composed of *Taxus chinensis*, red ginseng, and Gancao (*Glycyrrhiza uralensis* Fisch.), which have the functions of strengthening the vital Qi, dispelling pathogenic factors, promoting blood circulation, dissolving stagnation, and resolving hard lump, etc. Modern pharmacological studies have shown that all herbs or their active ingredients have antitumor and immunomodulation activities [20]. *Taxus chinensis* is the monarch herb of CTCC and possesses many components of taxanes, such as paclitaxel, 10-deacetylbaccatin III, baccatin III, and cephalomannine. Paclitaxel is the most important active component and has been widely used as an anticancer drug [20–22], which can suppress cancer cell mitosis and induce cell apoptosis [23]. The major active components of red ginseng are several ginsenosides, such as ginsenoside Rg1, Re, and Rb1, which can enhance immune function and suppress cancer cell proliferation. The major active ingredients of Gancao include glycyrrhizin, liquiritin, and glycyrrhetinic acid [24]. Glycyrrhizin can release glucuronic acid and combine with the poison containing carboxyl, hydroxyl groups to reduce the absorption of poisons, thus alleviating the toxic and side effects of drugs. Gancao contains Glycyrrhiza glucan, which can enhance the immune function of the body [25].

Both DCR and ORR are the key outcome measures for reporting the short-term clinical efficacy of anticancer therapy [26–28]. A total of 410 sufferers with NSCLC were included in our study. The pooled data of the current meta-analysis clearly suggested that CTCC added to chemotherapy could significantly improve the DCR (RR = 1.15, 95% CI: 1.06–1.25, *P* = 0.006) and ORR (RR = 1.38, 95% CI: 1.18–1.63, *P* *<* 0.00001); CTCC in combination with chemotherapy was significantly better than chemotherapy alone, indicating that CTCC might have synergic interactions with chemotherapy drugs and increase the sensitivity of chemotherapy.

QOL is an important clinical outcome of cancer patients undergoing chemotherapy, to improve QOL representing a main treatment goal [29–31]; therefore, it is defined as one of the secondary endpoints in this study. The meta-analysis result of QOL (MD = 8.69, 95% CI: 7.62–10.13, *P* < 0.006] revealed that the QOL of lung cancer patients treated with CTCC plus chemotherapy was significantly improved compared with chemotherapy alone (*P* < 0.00001). However, there were no trials that investigated the long-term effects of CTCC, such as survival rates of patients with lung cancer; therefore, it is necessary to perform more relevant clinical trials to evaluate more survival outcomes and further examine the long-term effectiveness of CTCC.

This meta-analysis has a few limitations: (a) only six studies were eligible and included in our systematic review and the overall quality of them was not high. Only two clearly described the method of randomization procedures [15, 18], and most did not distinctly depict the methods of randomization, concealment of allocation, or methods of blinding, which might have undermined the reliability of evidence to some extent. (b) The follow-up periods of the six included studies were relatively short, and no long-term outcomes were evaluated. (c) Due to the limited number of the included RCTs, it was infeasible to apply Egger's test or funnel plots to examine the possible publication bias. (d) Due to the insufficient individual data, sensitivity and subgroup analyses based on chemotherapy regimen, pathological type of lung cancer, or treatment duration, etc. were inadequate or infeasible.

## 5. Conclusion

Based on the available evidence, compared with chemotherapy alone, CTCC used as an adjunctive agent to chemotherapy for NSCLC can improve the clinical efficacy and quality of life and decrease the likelihood of adverse reactions, suggesting that CTCC might be an effective and safe adjunctive medicine to chemotherapy for NSCLC. However, considering the relatively small sample size and the inherent imperfections of the included randomized controlled trials, more high-quality clinical trials with longer follow-up time are needed to further assess the efficacy and safety of this combined treatment regimen.

## Figures and Tables

**Table 1:** Main features of the studies included in the current meta-analysis.

Reference	Design	Sample size (T/C)	Outcome measures	Intervention	Course of treatment	Cancer staging
Cao, 2016	RCT	29/29	DCR; ORR; KPS	T: CTCC + DP; C: DP	6 weeks	Advanced
Gong, 2018	RCT	36/36	DCR; ORR; QOL; adverse reaction rate	T: CTCC + GP; C: GP	12 weeks	Advanced
Li, 2017	RCT	42/42	DCR; ORR; tumor markers; Adverse reaction rate	T: CTCC + GP; C: GP	12 weeks	III, IV
Ren, 2018	RCT	38/38	DCR; ORR; KPS; tumor markers	T: CTCC + DP; CTCC + GP; CTCC + AP; C: DP; GP; AP	6 weeks	III, IV
Zhang H., 2017	RCT	30/30	DCR; ORR; adverse reaction rate; Immune function	T: CTCC + GP + Endostar; C: GP + Endostar	12 weeks	III, IV
Zhang X., 2015	RCT	30/30	DCR; ORR; KPS; maximum diameter of tumor; tumor markers	T: CTCC + TP/Paclitaxel plus nedaplatin; C: TP/Paclitaxel plus nedaplatin	8 weeks	II, III, IV

RCT**:** randomized controlled trial; T/C: treatment group/control group; CTCC: compound *Taxus chinensis* capsule; DCR**:** disease control rate; ORR: objective response rate; QOL: quality of life; KPS: Karnofsky health score; adverse reaction rate; tumor markers; immune function; Serum SIL-2R and IGF-1 levels; the area of cancer focal area; maximum diameter of tumor; GP: gemcitabine plus cisplatin; DP: docetaxel plus cisplatin; TC: paclitaxel plus carboplatin; TP: paclitaxel plus cisplatin; AP: pemetrexed plus cisplatin.

**Table 2:** The summary of the current meta-analysis results.

Outcome/subgroup		Number of studies	Number of participants	The selected statistical methods	Pooled effect size	*P*
DCR		6	410	RR (fixed), 95% CI	1.15 [01.06, 1.25]	0.0006^*∗*^
				OR (fixed), 95% CI	2.80 [1.54, 3.58]	0.0007^*∗*^
				RD (fixed), 95% CI	0.12 [0.06, 0.19]	0.0003^*∗*^
ORR		6	410	RR (fixed), 95% CI	1.15 [01.06, 1.25]	<0.0001^*∗*^
				OR (fixed), 95% CI	2.35 [1.54, 3.58]	<0.0001^*∗*^
				RD (fixed), 95% CI	0.19 [0.10, 0.27]	<0.0001^*∗*^
KPS		3	194	SMD (random), 95% CI	1.50 [1.17, 1.82]	<0.00001^*∗*^
				MD (fixed), 95% CI	8.69 [7.26, 10.13]	<0.00001^*∗*^
Adverse reactions	Gastrointestinal	4	274	RR (fixed), 95% CI	0.6[0.43, 0.86]	0.04^*∗*^
				OR (fixed), 95% CI	0.47[0.28, 0.78]	0.004^*∗*^
				RD (fixed), 95% CI	−0.17[−0.28, −0.06]	0.003^*∗*^
	Leukopenia	3	202	RR (fixed), 95% CI	0.44[0.27, 0.71]	0.0009^*∗*^
				OR (fixed), 95% CI	0.31[0.16, 0.60]	0.0006^*∗*^
				RD (fixed), 95% CI	−0.22[−0.34, −0.10]	0.0003^*∗*^
	Thrombocytopenia	2	144	RR (fixed), 95% CI	0.35[0.16, 0.78]	0.010^*∗*^
				OR (fixed), 95% CI	0.28[0.11, 0.71]	0.01^*∗*^
				RD (fixed), 95% CI	−0.18 [−0.30, −0.06]	0.004^*∗*^
	Fever	2	144	RR (fixed), 95% CI	0.28 [0.11, 0.71]	0.007^*∗*^
				OR (fixed), 95% CI	0.22 [0.08, 0.64]	0.005^*∗*^
				RD (fixed), 95% CI	−0.18 [−0.29, -0.07]	0.002^*∗*^
	Rash	2	144	RR (fixed), 95% CI	0.48 [0.26, 0.87]	0.02^*∗*^
				OR (fixed), 95% CI	0.37 [0.17, 0.82]	0.01^*∗*^
				RD (fixed), 95% CI	−0.18 [−0.32 −0.04]	0.01^*∗*^

^*∗*^Favours the experimental group with statistical significance. DCR, disease control rate; RR, relative ratio; OR, odds ratio; CI, confidence intervals; RD, risk difference; ORR, objective response rate; KPS: Karnofsky health score; SMD, standardized mean difference; MD, mean difference; QOL, quality of life; WMD, weighted mean difference.

## Data Availability

All data are included within the article.

## Abbreviations

CBM: Chinese biomedicine database
CI: Confidence interval
CNKI: Chinese National Knowledge Infrastructure
CR: Complete response
CTCC: Compound *Taxus chinensis* capsule
KPS: Karnofsky performance status
DCR: Disease control rate
MD: Mean difference
NSCLC: Non-small-cell lung cancer
NC: No change
ORR: Objective response rate
OR: Odds ratio
PR: Partial response
PD: Progressive disease
QOL: Quality of life
RD: Risk difference
RCT: Randomized controlled trial
RR: Risk ratio
SD: Stable disease
SMD: Standardized mean difference
TCM: Traditional Chinese medicine
WHO: World Health Organization
WMD: Weighted mean difference.

## Supplementary Materials

Supplementary Materials The appendix represents the search strategy of PubMed with the PICO model.
