# Supplementary material for: Compound Taxus chinensis Capsule Combined with Chemotherapy for Non-Small-Cell Lung Cancer: A PRISMA-Compliant Systematic Review and Meta-Analysis of Randomized Controlled Trials
Source: Evid Based Complement Alternat Med. 2021 Dec 16;2021:9535061. doi: 10.1155/2021/9535061 (PMC8702306; doi:10.1155/2021/9535061)
Supplement: Supplementary Materials — The appendix represents the search strategy of PubMed with the PICO model. [file 9535061.f1.docx]

**Appendix**

**The search strategy of Pubmed with PICO model**

| **PICO ELEMENTS** | **KEYWORDS** | **SEARCH TERMS** | **SEARCH STRATEGIES** |
| --- | --- | --- | --- |
| P  (Patient or Population) | Patients with non-small-cell lung cancer | non-small-cell lung cancer | Non-small-cell lung cancer  OR  Non-small-cell lung carcinoma  OR  Non-small-cell lung carcinomas  OR  Non-small-cell lung  OR  Lung cancer, non-small-cell  OR  Lung carcinomas, non-small-cell  OR  Lung carcinomas, non-small-cell  OR  Cancer, non-small cell lung  OR  Carcinoma, non-small cell lung |
| I  (Intervention) | CTCC plus chemotherapy | Compound Taxus Chinensis capsule | Compound Taxus Chinensis capsule  OR  Compound Hongdoushan Capsule |
| C  (Comparison) | Chemotherapy alone | Chemotherapy | Chemotherapy  OR  Chemotherapeutics  OR  chemotherapeutic agents  OR  Chemotherapeutic drugs |
| O  (Outcome) | Disease control rate (DCR), objective response rate (ORR), adverse reactions, and quality of life | Disease control rate (DCR), objective response rate (ORR), adverse reactions, and quality of life | Disease control rate (DCR)  OR  Objective response rate (ORR)  OR  Efficacy  OR  Effectiveness  OR  Efficiency  OR  Adverse reactions  OR  Toxicity  OR  Toxicities  OR  Toxic effects  OR  Safety  OR  Quality of life  OR  Karnofsky score |
